# Identification of prognostic risk model based on plasma cell markers in hepatocellular carcinoma through single-cell sequencing analysis

**DOI:** 10.3389/fgene.2024.1363197

**Published:** 2024-05-27

**Authors:** Yuanqi Li, Hao Huang, Qi Wang, Xiao Zheng, Yi Zhou, Xiangyin Kong, Tao Huang, Jinping Zhang, You Zhou

**Affiliations:** ^1^ Tumor Biological Diagnosis and Treatment Center, The Third Affiliated Hospital of Soochow University, Changzhou, China; ^2^ Jiangsu Engineering Research Center for Tumor Immunotherapy, Changzhou, China; ^3^ Institute of Cell Therapy, Soochow University, Changzhou, China; ^4^ CAS Key Laboratory of Tissue Microenvironment and Tumor, Shanghai Institute of Nutrition and Health, Chinese Academy of Sciences, Shanghai, China; ^5^ Bio-Med Big Data Center, Shanghai Institute of Nutrition and Health, Chinese Academy of Sciences, Shanghai, China; ^6^ Institutes of Biology and Medical Sciences, Soochow University, Suzhou, China

**Keywords:** single-cell RNA sequencing, RNA, tumor-infiltrating B lymphocytes, plasma cells, hepatocellular carcinoma, prognostic risk model, therapeutics

## Abstract

Hepatocellular carcinoma (HCC) represents a substantial global health burden. Tumorinfiltrating B lymphocytes (TIL-Bs) contribute to tumor progression and significantly impact the efficacy of tumor therapy. However, the characteristics of TIL-Bs in HCC and their effect on HCC therapy remain elusive. Single-cell RNA sequencing (scRNAseq) was applied to investigate the heterogeneity, cellular differentiation and cell-cell communication of TIL-Bs in HCC. Further, the Cancer Genome Atlas-liver hepatocellular carcinoma (TCGA-LIHC) and liver cancer institutes (LCI) cohorts were applied to construct and validate the plasma cell marker-based prognostic risk model. The relationship between the prognostic risk model and the responsiveness of immunotherapy and chemotherapy in patients with HCC were estimated by OncoPredict and tumor immune dysfunction and exclusion (TIDE) algorithm. Finally, we established nomogram and calibration curves to evaluate the precision of the risk score in predicating survival probability. Our data identified five subtypes of TIL-Bs in HCC, each exhibiting varying levels of infiltration in tumor tissues. The interactions between TIL-Bs and other cell types contributed to shaping distinct tumor microenvironments (TME). Moreover, we found that TIL-Bs subtypes had disparate prognostic values in HCC patients. The prognostic risk model demonstrated exceptional predictive accuracy for overall survival and exhibited varying sensitivities to immunotherapy and chemotherapy among patients with HCC. Our data demonstrated that the risk score stood as an independent prognostic predictor and the nomogram results further affirmed its strong prognostic capability. This study reveals the heterogeneity of TIL-Bs and provides a prognostic risk model based on plasma cell markers in HCC, which could prove valuable in predicting prognosis and guiding the choice of suitable therapies for patients with HCC.

## Introduction

Hepatocellular carcinoma (HCC), representing a significant majority of liver cancer cases, stands as a major concern for global public health (Petrick and McGlynn, 2019; Sung et al., 2021). Increasing evidence underlines the essential role performed by complex tumor microenvironment (TME) in impacting the effectiveness of tumor therapies and promoting tumor progression (Pitt et al., 2016; Bejarano et al., 2021). B cells and plasma cells, commonly known as tumor-infiltrating B lymphocytes (TIL-Bs), constitute a significant component of TME (Wouters and Nelson, 2018). TIL-Bs are increasingly recognized as pivotal players in modulating tumor responses, thereby making them promising targets for therapeutic interventions (Yuen et al., 2016). However, the heterogeneity of TIL-Bs determines their dual role in anti-tumor immunity (Laumont et al., 2022; Nakamura et al., 2023). TIL-Bs actively participate in cellular immunity by engaging in antigen presentation and fostering the development of tertiary lymphoid structures (TLS). As a result, this amplifies anti-tumor immune responses and increases the efficacy of immune checkpoint inhibitor (ICI) treatment (Helmink et al., 2020). Furthermore, plasma cells have long been acknowledged for their capacity to generate antibodies, contributing to the elimination of tumor cells through antibody-mediated cellular cytotoxicity (Kurai et al., 2007). Simultaneously, B cells may also be implicated in promoting tumor progression. Regulatory B (Breg) cells, characterized by their secretion of IL10 and IL35, primarily exert negative immune regulation (Michaud et al., 2021). Additionally, antibodies can generate circulating immune complexes (CICs), which accelerates tumor progression. Moreover, the function of TIL-Bs vary across different tumor types (Andreu et al., 2010; Jiang et al., 2020; Li et al., 2020). To date, the characteristics of TIL-Bs in HCC and their impact on prognosis and therapy efficacy of HCC patients have not been established.

Single-cell RNA sequencing (scRNA-seq) technology has emerged as a potent instrument for unveiling the complexities of TME, illuminating previously uncharted territories (Lei et al., 2021). In contrast to bulk RNA-seq data, which can solely gauge overall gene expression levels in entire tissue specimens, scRNA-seq has the capability to identify genes that are distinctly highly expressed in specific cell subtypes (Shaul et al., 2021). Furthermore, scRNA-seq facilitates the examination of cell-to-cell interactions within the TME and the exploration of dynamic processes related to cellular differentiation and development (Armingol et al., 2021; Derakhshan et al., 2022). This technology enables a comprehensive analysis of the roles played by specific cell subtypes in tumor progression. On the other hand, bulk RNA-seq data offer larger sample sizes and clinical information, thus bridging the gap between basic research and clinical applications. Therefore, the incorporation of single cell and bulk RNA sequencing appears encouraging in comprehensively unraveling the heterogeneity of TIL-Bs and exploring their relationship with clinical outcome.

In this study, we combined single-cell and bulk RNA-sequencing analyses to investigate the heterogeneity of TIL-Bs in HCC and their relationship with patient prognosis. Our data systematically delineated the landscape of TIL-Bs and their intercellular communications with other constituents within the HCC TME. We additionally evaluated the prognostic significance of TIL-Bs subgroups in the Cancer Genome Atlas-liver hepatocellular carcinoma (TCGA-LIHC) and liver cancer institutes (LCI) cohorts. Furthermore, we constructed a plasma cell marker-based prognostic risk model, which correlated with the response of HCC patients to both immunotherapy and chemotherapy.

## Materials and methods

### Sample collection and processing

From May 2021 to November 2022, eight tissue samples in total were gathered at The Third Affiliated Hospital of Soochow University, including four postoperative HCC tissues and four normal liver tissues (three of which were paired). The clinical characteristics of these HCC patients were provided in Supplementary Table S1. The study was conducted with approval from the Ethical Committee of the hospital, and all participants had provided their informed consent. The resected tissues were cut into approximately 0.125 cm^3^ pieces and immediately immersed in a 4°C Tissue Storage Solution (Miltenyi, 130-100-008). Single-cell suspension was prepared according to the manufacturer’s instructions of 10X Genomics (https://www.10xgenomics.com/support/single-cell-immune profiling/documentation/steps/sample-prep). And single-cell suspension that met the required criteria were subjected to 5′single-cell RNA sequencing.

### ScRNA-seq data processing

Cell Ranger software (version 5.0.0) and the GRCh38 Reference Genome (2020) were used for creating the raw gene expression matrix for further analysis. Quality control, normalization, dimension reduction and clustering were performed by Seurat (version 4.1.0) package. R package DoubletFinder (version 2.0.3) was utilized to identify and remove doublets based on default parameters to ensure the inclusion of high-quality cells. Cells exhibiting in excess of 5,000 unique molecular identifier (UMI) counts and mitochondrial gene counts exceeding 10% were excluded from the analysis. And 3,000 highly variable genes were selected for following evaluation. Further, R package Harmony (version 0.1.0) was applied to address the batch effects. Top 30 principal components were retained based on the Elbow plot function in Seurat. The FindClusters function in Seurat was used to identify the main cell clusters, with a resolution set at 0.2. Uniform manifold approximation and projection (UMAP) was used for visualization of cell clusters. Known biological cell types were assigned to each cell based on conventional markers.

### Data acquisition

The scRNA-seq dataset, comprising eight normal samples and ten tumor samples, was retrieved from the Gene Expression Omnibus (GEO) database using the access code GSE149614. We obtained transcriptome data and clinical information for 356 tumor samples in the TCGA-LIHC cohort from the University of California, Santa Cruz (UCSC) Xena database, available at https://xenabrowser.net/datapages/. The expression matrix and clinical information of the LCI cohort, comprising 225 tumor tissues collected from Zhongshan Hospital of Fudan University, were downloaded from GSE14520. The somatic mutation data of TCGA-LIHC was downloaded from TCGA database.

### Gene set enrichment analysis

R package AUCell (version 1.16.0) was employed to estimate the area under curve (AUC) score of germinal center (GC) B cells and plasma cells. The gene set of GC B cells and plasma cells were obtained from the highly expressed genes of an ex vivo-sorted human GC B cells and plasma cells (GSE12366). Another GC B cells signature genes (AICDA, BCL6, POLH, P2RY8, SEMA4A, FOXO1, BACH2, BATF, CD86, IRF8, DOCK8) were obtained from Cui et al. (2021) study. The enrichment score of TLS was evaluated by ssGSEA function in R package Gene Set Variation Analysis (GSVA) (version 1.42.0). Signature genes for TLS were identified in a previous study, including CD79B, CD1D, CCR6, LAT, SKAP1, CETP, EIF1AY, RBP5, and PTGDS (Cabrita et al., 2020).

### Trajectory analysis of TIL-Bs

The trajectory of TIL-Bs was analyzed by R package Monocle2 (version 2.22.0). We selected variable features identified in Seurat to order cells along the trajectory. Pseudotime analysis was employed to infer the developmental progression of TIL-Bs. To identify genes that exhibited branch-dependent expression, we applied Branched Expression Analysis Modeling (BEAM) analysis. Furthermore, the plot_genes_branched_heatmap function was utilized to visualize genes that were significantly branch dependent.

### GO and KEGG enrichment analysis

ClusterProfile (version 3.14.3) was applied for the purpose of conducting enrichment analysis for both Gene Ontology (GO) and Kyoto Encyclopedia of Genes and Genomes (KEGG) pathways. Significance was determined based on an adjusted *p*-value calculated using the false discovery rate (FDR) method, with a threshold set at less than 0.05.

### Recognition of cell-cell communications

R package CellChat (version 1.5.0) was harnessed for exploring the crosstalk and interconnections between TIL-Bs and other cellular components within the complex TME of HCC. The ligand-receptor interaction database, Secreted Signaling, was chosen for subsequent analysis. Cell-cell communication networks were inferred using standard procedures and default parameters. CellChat object from tumor data and normal data was constructed separately, and then merged them together. The netVisual_heatmap function in CellChat was utilized to identify differential numbers of interactions or interaction strengths among different cell populations across the tumor dataset and normal dataset. Additionally, the netVisual_bubble function was applied to identify upregulated signaling ligand-receptor pairs in tumor tissues.

### Estimation of infiltration of naïve B cells, memory B cells and plasma cells

R package cell-type identification by estimating relative subsets of RNA transcripts (CIBERSORT) (version 0.1.0) was utilized for estimating the proportion of TIL-Bs subtypes infiltration in bulk RNA data. TCGA-LIHC and LCI cohorts were used as input expression matrices and the LM22 gene expression matrix was used as a reference for running CIBERSORT.

### Survival analysis

Survival analysis was conducted with R package Survival (version 3.2.13), and the Cox proportional hazards model was utilized to determine hazard ratios (HR) for the assessment of the association between factors and survival outcomes. Kaplan-Meier survival curves were generated and modeled using the Survfit function. To identify the ideal cutoff point for patient stratification into two groups, R package Survminer (version 0.4.9) was utilized.

### Construction of plasma cells associated genes prognostic model

Plasma cells associated genes were recognized by FindAllMarkers function of Seurat package under the parameters of onlypo = T, minper = 0.2. We filtered out genes related to prognosis through univariate Cox regression analysis, followed by subjecting the risk genes to least absolute shrinkage and selection operator (LASSO) analysis for further refinement. We selected the λ value associated with the minimum partial likelihood deviance as the optimal λ in our investigation. Subsequently, six genes were selected for constructing the prognostic risk model through multivariate Cox regression analyses, utilizing the following formula: riskscore = SEC61A1_exp_*0.000811515+DNAJC1_exp_*0.009154278+EIF5B_exp_*0.012715447+DNAJB4_exp_*0.018562242+ST6GALNAC4_exp_*0.013184291+CCDC88A_exp_*0.009150427, where gene_exp_ was the expression level of the gene. Subsequently, patients were stratified into high-risk and low-risk categories based on the median risk score.

### Drug-sensitivity analysis and tumor immune dysfunction and exclusion (TIDE) analysis

The investigation of drug sensitivity in TCGA-LIHC cohort was carried out using R package OncoPredict (version 0.2). The information of Genomics of Drug Sensitivity in Cancer (GDSC) was downloaded from oncoPredict (https://osf.io/c6tfx/). The half maximal inhibitory concentration (IC50) of each HCC sample was estimated by calcPhenotype function in oncoPredict package with default parameters. Subsequently, the IC50 values of common drugs for HCC treatment were compared between two risk groups. Additionally, potential immunotherapeutic response of patients in the TCGA-LIHC cohort was assessed using the TIDE tool, which is accessible at http://tide.dfci.harvard.edu/.

### Valuation of predicted accuracy of the risk score

The assessment of whether the risk score demonstrated independent predictive capacity for patient prognosis was based on univariate and multivariate Cox regression analyses. Additionally, a Nomogram analysis was constructed using R package rms (version 6.5.0).

### Statistical analysis

All statistical analyses were conducted using the R software (version 4.1.2). Comparisons between the two groups were executed using the Wilcoxon test. The assessment of the proportion of immunotherapy response between the different risk groups was accomplished through Chi-squared test. *p* < 0.05 was deemed as statistically significant.

## Results

### ScRNA-seq landscape of HCC patients

**FIGURE 1:**
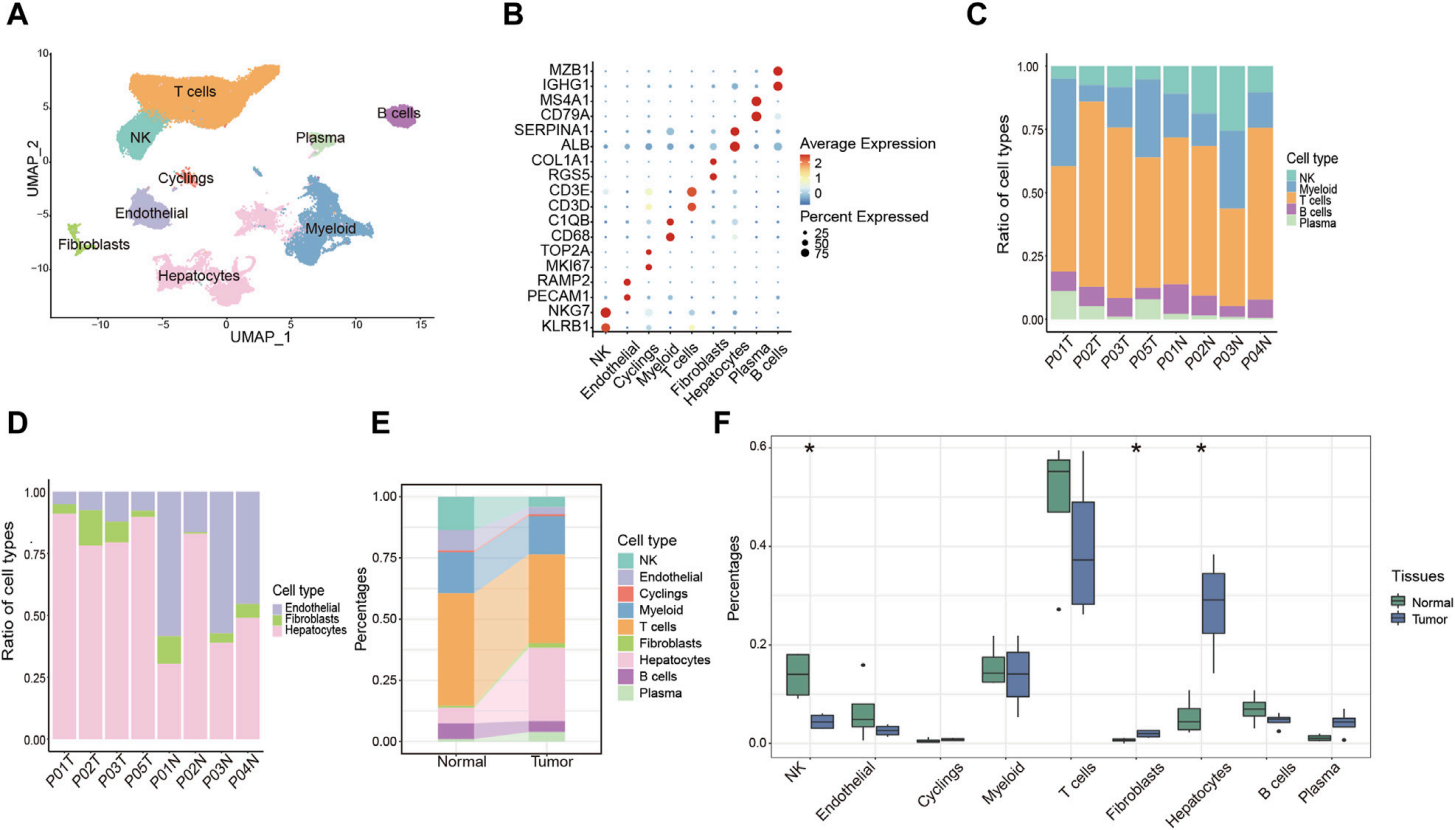
Overview of the single-cell landscape for HCC patient. **(A)** UMAP analysis of clustering single cells colored by cell types. **(B)** Dot plot showing the expression of conventional markers of each cell type. **(C,D)** Barplot showing the composition of cell types in immune cells **(C)** and components of non-immune cells **(D)** among each sample. **(E)** Alluvial diagram showing percentages of each cell type between tumor and normal liver tissues. **(F)** Percentages of each cell type between tumor and normal liver tissues. *p < 0.05.

To systematically investigate the TME of HCC patients, we performed 10X genomic scRNA-seq of collecting HCC samples and a total of 73,126 cells were obtained after quality control. Utilizing established gene markers as references, we successfully delineated nine distinct cell populations, including five immune cell clusters (T cells, myeloid cells, natural killer (NK) cells, plasma cells and B cells), three non-immune cell clusters (endothelial cells, hepatocytes and fibroblasts), and one proliferating cell cluster designated as “Cyclings” (Figures 1A, B). Furthermore, we proceeded to investigate the cellular composition within each sample. Particularly, T cells were the predominant immune cell population, present in both tumor and adjacent normal liver tissues (Figure 1C). Within non-immune cell populations, hepatocytes were the predominant cell type within tumor tissues, whereas endothelial cells were more prevalent in normal liver tissues (Figure 1D). Furthermore, the data indicated a notable reduction of NK cell and a considerable increase of fibroblasts and hepatocytes in tumor tissues (Figures 1E, F). Moreover, a conspicuous trend was observed with an upregulation of plasma cells and a downregulation of B cells within tumor tissues (Figures 1E, F). Taken together, these findings collectively provide a comprehensive overview of the TME in HCC patients.

### Single-cell atlas of TIL-Bs in HCC

**FIGURE 2:**
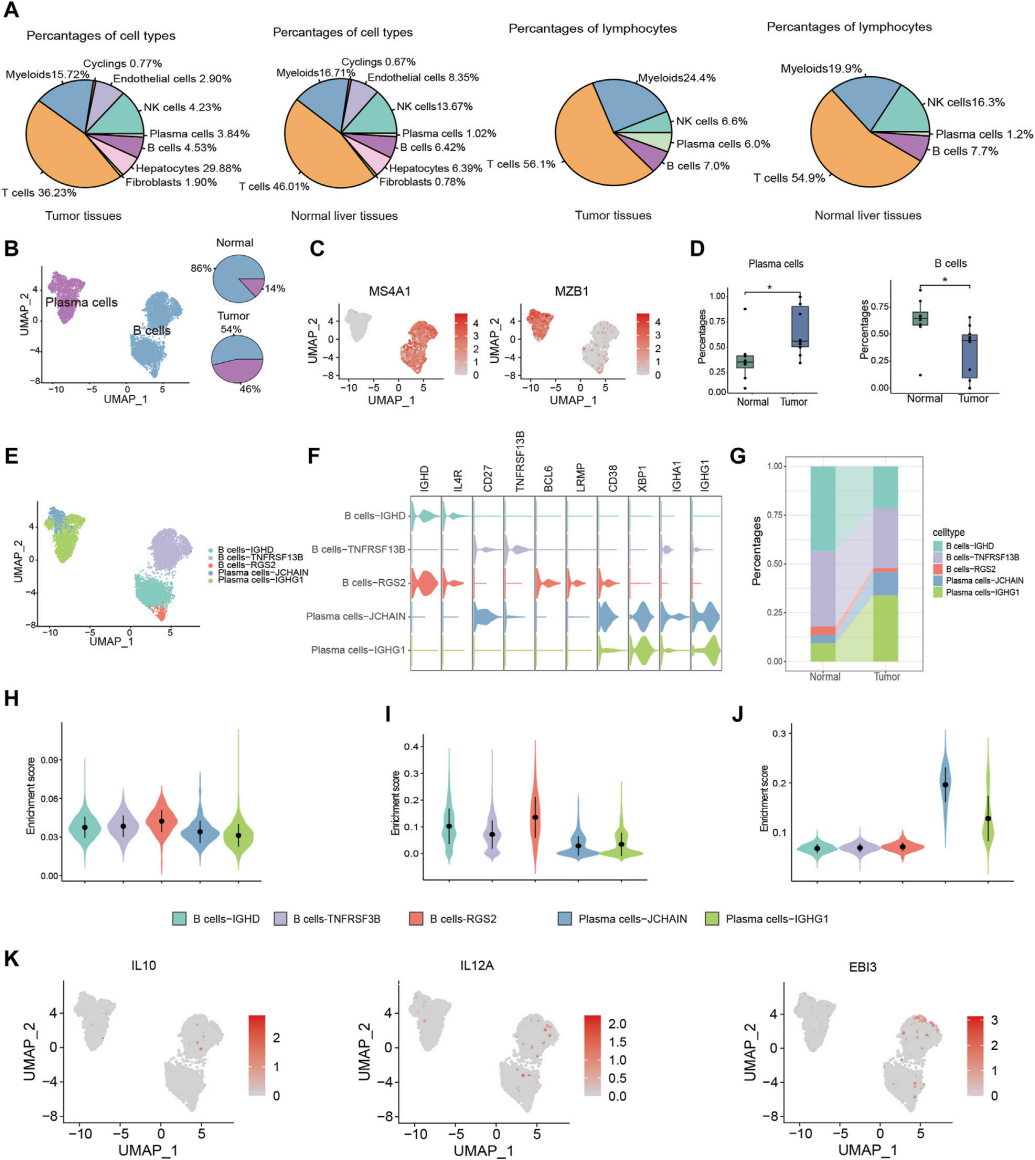
Distinctive characteristics of TIL-Bs subtypes in TME of HCC. **(A)** The population of B cells and plasma cells in tumor tissues and normal liver tissue. **(B)** UMAP plot identifying the clusters of plasma cells and B cells (left) and pie plot showing the composition of TIL -Bs in tumor and normal tissue (right) in TME of HCC. **(C)** Feature plot showing the expression of MS4A1 and MZB1. **(D)** Boxplot showing the alteration of B cells and plasma cells between normal and tumor tissues according to GSE149614 cohort. **(E)** UMAP showing the subtypes of TIL-Bs at high resolution. **(F)** The expression of selected genes among subtypes of TIL-Bs. **(G)** Alluvial diagram showing percentages of subtypes of TIL-Bs between tumor and normal liver tissues. **(H)** Enrichment score of an ex vivo-sorted human GC B cells marker genes among TIL-Bs subtypes. **(I)** Enrichment score of GC B cells marker genes in Cui et al. (2021) study among TIL-Bs subtypes. **(J)** Enrichment score of an ex vivo-sorted human plasma cells marker genes among TIL-Bs subtypes. **(K)** The expression of regulatory B (Breg) cells marker genes via feature plot. *p < 0.05.

TIL-Bs, being a critical element within the immune cell population, wield a central influence in anti-tumor responses. To explore the role of TIL-Bs in the HCC TME, we initially investigated the population of B cells and plasma cells in both tumor tissues and normal liver tissues. In tumor tissues, B cells and plasma cells comprise 4.53% and 3.84% of total cells, respectively, and 7.0% and 6.0% of lymphocytes. In contrast, in normal liver tissues, B cells and plasma cells account for 6.42% and 1.02% of total cells, respectively, and 7.7% and 1.2% of lymphocytes (Figure 2A). Next, we conducted an independent analysis of TIL-Bs to elucidate their heterogeneous characteristics. Firstly, TIL-Bs were categorized into B cells and plasma cells according to the expression of MS4A1 and MZB1 (Figures 2B, C). In normal liver tissues, B cells constituted 86% of the TIL-Bs population, while plasma cells comprised 14%. In contrast, the distribution was altered in tumor tissues, with 54% represented by B cells and 46% by plasma cells, indicating a potential proclivity for B cells within the tumor tissues to differentiate into plasma cells (Figure 2B). To validate these observations concerning TIL-Bs alterations in tumor tissues, we expanded our analysis to a larger cohort encompassing eight normal liver tissues and ten tumor tissues (Lu et al., 2022). As anticipated, there was a substantial increase of plasma cells, while a significant reduction of B cells in tumor tissues (Figure 2D).

To comprehensively characterize the TIL-Bs subpopulations within the HCC TME, we further subclustered them at a higher resolution, leading to the identification of two plasma cell clusters (Plasma cells-JCHAIN and Plasma cells-IGHG1) and 3 B cell clusters (B cells-IGHD, B cells-TNSF13B, and B cells-RGS2) (Figure 2E). B cells-IGHD cluster exhibited elevated expression of IGHD, suggesting its potential identity as a population of naïve B cells. The B cells-TNSF13B cluster displayed higher levels of CD27 and TNSF13B, indicative of a memory phenotype. The B cells-RGS2 cluster exhibited elevated expression levels of BCL6 and LRMP, indicating the germinal center (GC) B cells phenotype (Figure 2F). Meanwhile, the Plasma cells-IGHG1 and Plasma cells-JCHAIN clusters displayed high expression levels of IGHG1 and IGHA1, respectively, suggesting their potential role in secreting IgG and IgA antibodies (Figure 2F). Furthermore, we observed an increased proportion of both Plasma cells-IGHG1 and Plasma cells-JCHAIN in tumor tissues (Figure 2G).

To confirm the precision of phenotype identification using conventional markers, we conducted gene set enrichment analysis. The gene sets for GC B cells were derived from genes with high expression levels in flow sorting human GC B cells and Cui et al. (2021)’s study. And the gene set for plasma cells was derived from highly expressed genes in ex vivo-sorted plasma cells. The enrichment scores for both B cell gene sets were found to be highest in the B cells-RGS2 cluster (Figures 2H, I). Similarly, Plasma cells-JCHAIN and Plasma cells-IGHG1 exhibited higher enrichment score for ex vivo-sorted human plasma cell marker genes relative to the other 3 B cell clusters (Figure 2J). These data underscored the precision of our TIL-B subpopulation classification. Notably, Breg cells, known for their immunosuppressive functions in various cancers, are typically characterized by the expression of effector molecules such as IL10 and IL35 (encoded by IL12A and EBI3) (Downs-Canner et al., 2022). However, in line with recent single-cell sequencing studies, we did not identify Breg cells based on the expression of IL10 and IL35 (Figure 2K) (Hu et al., 2021), which might due to the expression discrepancy between protein and RNA.

### Dynamic gene expression during B cell differentiation

**FIGURE 3:**
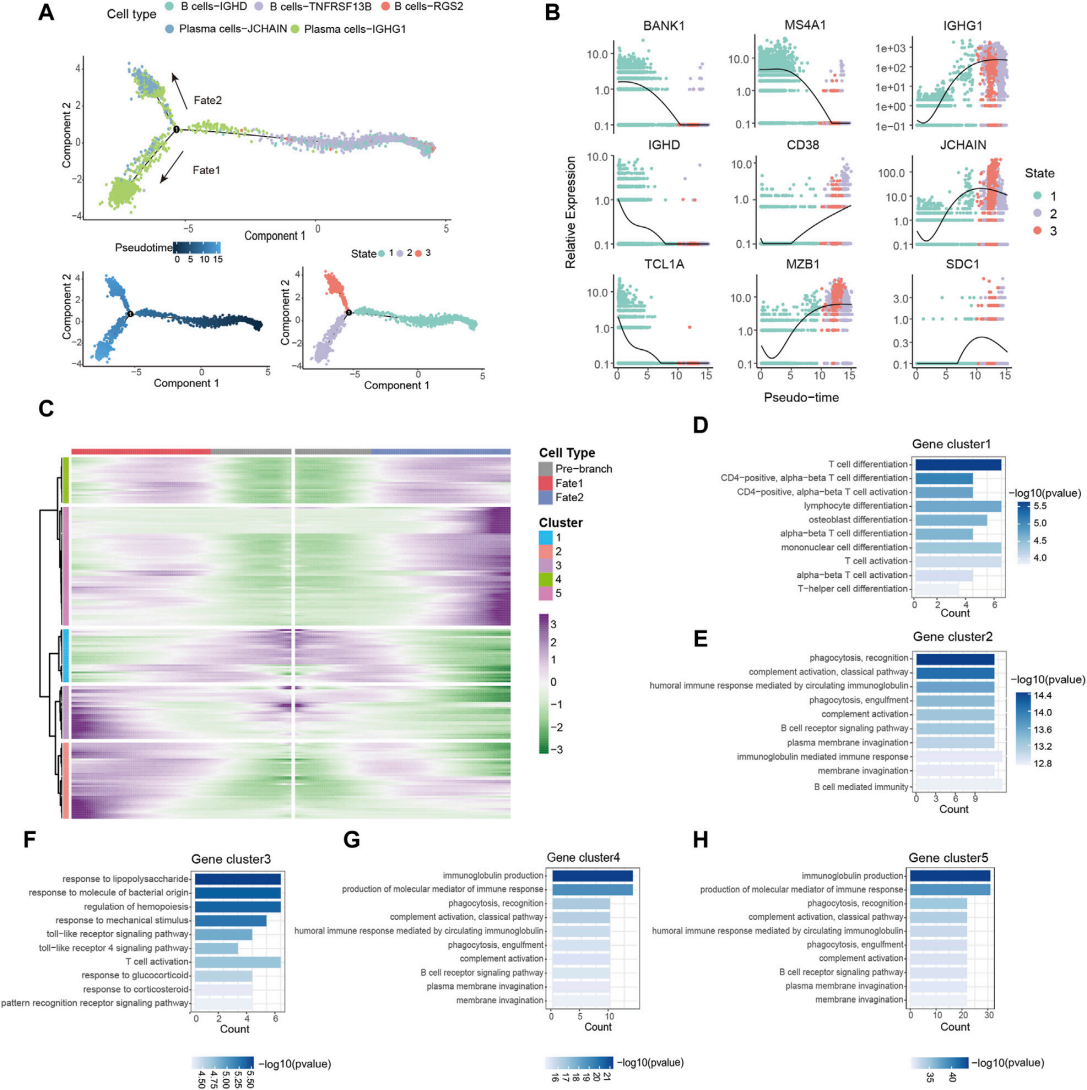
Dynamic gene expression in the differentiation of B cells. **(A)** Pseudo-trajectory of TIL-Bs colored by cell types, pseudo-time and state. **(B)** Expression of select genes during pseudo-time colored by state. **(C)** Heatmap showing the modules of gene clusters in two cell fates of the branch point. **(D–H)** GO function analysis of each gene cluster in **(C)**.

Based on the observed upregulation of plasma cells in tumor tissues (Figure 2), we hypothesized that B cells might be forced to differentiate into plasma cells under the pressure of TME. To investigate the differentiation trajectories of TIL-Bs, we employed pseudotime analysis using the Monocle2. This analysis identified three distinct states of TIL-Bs. “State 1,” representing the initial phase of differentiation, was predominantly composed of B cells, while “State 2” and “State 3” were primarily comprised of the Plasma cells-IGHG1 and Plasma cells-JCHAIN clusters, respectively (Figure 3A). Subsequently, the expression patterns of selected genes were scrutinized within these three states. We observed a gradual downregulation of genes specific to B cells, such as BANK1, IGHD1, TCL1A and MS4A1, whereas genes characteristic of plasma cells, including CD38, MZB1, IGHG1, JCHAIN and SDC1, exhibited an upregulation trend (Figure 3B).

To conduct a thorough investigation of gene expression profiles linked with B cell differentiation, we employed branched expression analysis modeling (BEAM) analysis, a statistical method capable of discerning gene expression changes based on branch points (Trapnell et al., 2014). BEAM analysis revealed that during the differentiation of B cells, “gene cluster 1” exhibited a downregulation, while gene “clusters 2,” “clusters 4” and “clusters 5” displayed a gradual upregulation (Figure 3C). The gene set of each gene cluster was provided in Supplementary Table S2. Remarkably, “gene cluster 3” exhibited an upregulation during B cell differentiation into the Plasma cells-IGHG1 cluster but a downregulation during differentiation into the Plasma cells-JCHAIN cluster (Figure 3C).

GO enrichment analysis was carried out with the aim of acquiring insights into the biological roles of these gene clusters. All results from the GO enrichment analysis were available in Supplementary Table S3. We observed enrichment of CCR7, GPR183 and LY9 in “gene cluster 1,” which are associated with T cell differentiation and activation (Figure 3D; Supplementary Table S2). Notably, the downregulated expression of these genes has been linked to the induction of T follicular helper cells differentiation and their maintenance in the GC (Moschovakis et al., 2012; De Salort et al., 2013; Suan et al., 2015). Since the differentiation and development of B cells in the GC require the assistance of T follicular helper cells (Kräutler et al., 2017), B cells may differentiate into plasma cells in a T cell-dependent manner by downregulating the expression of these genes. “Gene clusters 2,” “gene clusters 4” and “gene clusters 5” were enriched in processes related to immunoglobulin production, complement activation, and phagocytosis, respectively (Figures 3E, G, H). Additionally, the transition of B cells into the Plasma cells-IGHG1 cluster was accompanied by the upregulation of genes associated with the toll-like receptor signaling pathway (Figure 3F). In summary, these findings provide evidence of dynamic gene expression patterns during B cells differentiation within the TME.

### Cell-cell interactions between TIL-Bs and other cell types

The functionality of immune cells hinges significantly on intercellular communication. Consequently, it is imperative to investigate the cell-cell interactions involving TIL-Bs and other cell types to elucidate the role of TIL-Bs in anti-tumor immunity. To achieve a higher resolution of cell type, we initially subdivided T cell and myeloid cell clusters into subgroups. We delineated seven distinct T cell subgroups according to the most expressed genes of each cluster and functional genes of T cells (Supplementary Figures S1A, B). We noted that the CD8-XCL1 cluster displayed elevated expression levels of cytotoxic genes (IFNG, GZMB, GZMH, PRF1 and NKG7) as well as genes associated with exhaustion (HAVCR2, TNFRSF9, TOX2, TIGIT and LAG3), indicative of exhausted T cells (Supplementary Figure S1B). Further, we found that CD4-FOXP3 cluster exhibited enrichment in tumor tissues (Supplementary Figure S1C) and manifested heightened expression of both immune co-stimulatory and immune co-inhibitory factors (Supplementary Figures S1D, E). Likewise, we classified myeloid cells into seven distinct subgroups based on conventional marker genes (Supplementary Figures S2A, B). Notably, the Macrophages-SPP1 cluster displayed enrichment in tumor tissues when contrasted with normal liver tissues (Supplementary Figure S2C). The Monocytes-S100A8 cluster exhibited high expression levels of genes associated with myeloid-derived suppressor cells (MDSC) (Supplementary Figure S2D), while the Macrophages-SPP1 and Macrophages-C1QC clusters expressed high levels of genes associated with tumor-associated macrophages (TAMs) (Supplementary Figure S2E).

**FIGURE 4:**
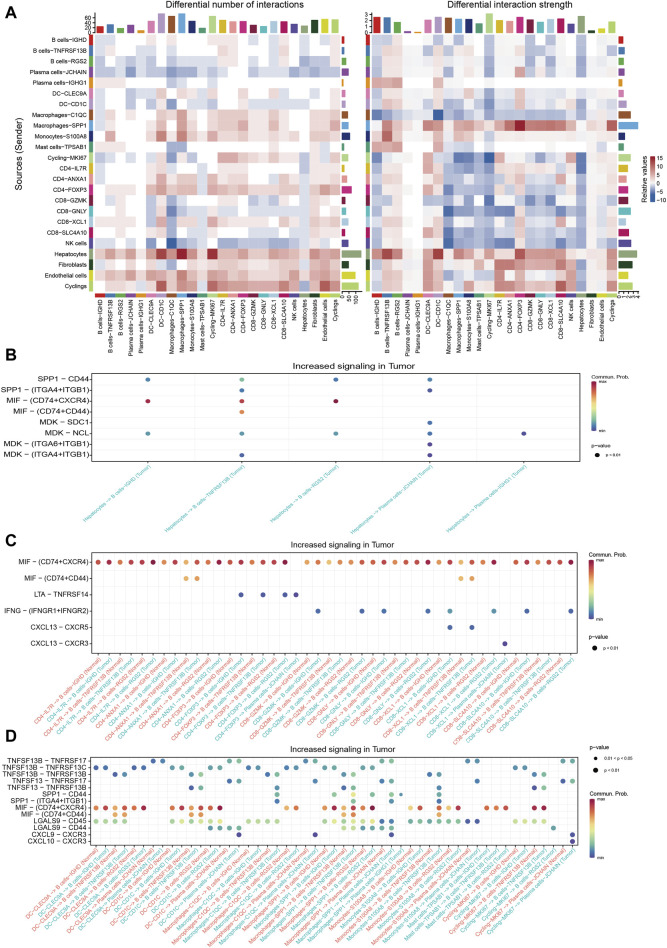
Cellular communications between TIL-Bs and other cell types in HCC. **(A)** Heatmap depicting the differential number of interactions or interaction strength between tumor tissues and normal liver tissues. Red indicates increased signaling in tumor tissues, while blue indicates decreased signaling in tumor tissues. **(B)** Dot plot showing the increased signaling ligand-receptor pairs between hepatocytes and TIL-Bs in tumor tissues. **(C)** Dot plot demonstrating the increased signaling ligand-receptor pairs between T cells and TIL-Bs in tumor tissues. **(D)** Dot plot illustrating the increased signaling ligand-receptor pairs between myeloid cells and TIL-Bs in tumor tissues.

Cellchat was utilized to probe the communications between TIL-Bs and other compositions in HCC TME. We integrated CellChat objects from tumor tissues and normal liver tissues to examine the differences in TIL-Bs’ cellular communication intensity between tumor and normal liver tissues. We observed that, in comparison to normal tissue, both the number and strength of interactions between hepatocytes and TIL-Bs were significantly increased in tumor tissues (Figure 4A). To investigate the potential mechanisms of TIL-Bs in the HCC TME, we focused on the upregulated signaling pathways within the tumor tissue. We did not identify signaling pathways through which TIL-Bs act on hepatocytes. However, hepatocytes could interact with TIL-Bs through the SPP1-CD44 and MIF-CD74/CXCR4 pathways (Figure 4B), both of which have been shown to be associated with the progression of HCC(Wirtz et al., 2021; He et al., 2023).

The interaction between T cells and TIL-Bs has been demonstrated to be associated with the prognosis and treatment response of cancer patients (Schina et al., 2023). Therefore, we further investigated the upregulated signaling pathways between T cells and TIL-Bs in HCC tumor tissues. When acting as sender cells, B cells-TNFRSF13B and Plasma cells-JCHAIN could activate CD40-FOXP3 and CD8-XCL1 through TNFSF9-TNFRSF9 signaling (Supplementary Figure S3A). Additionally, CD8-XCL1 could recruit B cells-IGHD and B cells-TNFRSF13B via the CXCL13-CXCR5 signaling pathway (Figure 4C), which is crucial for the formation of tertiary lymphoid structures (Tokunaga et al., 2019).

The interaction between myeloid cells and TIL-Bs has also been shown to be closely associated with the differentiation and function of TIL-Bs in the HCC TME (Wei et al., 2019). Our study revealed that Plasma cells-JCHAIN interacted with Macrophages-SPP1 through GAS-MERTK (Supplementary Figure S3B), a pathway known to be associated with tumor progression (Du et al., 2021). Additionally, macrophages and dendritic cells (DCs) could interact with TIL-Bs through the TNFSF13B-TNFRSF13B/TNFRSF13C and TNFSF13-TNFRSF13B signaling pathways (Figure 4D), which are pivotal for B cells’ differentiation into plasma cells. Moreover, Plasma cells-JCHAIN could be recruited by DC-CD1C, Macrophages-C1QC and Macrophages-SPP1 through CXCL9-CXCR3 (Figure 4D).

### Clinical prognosis of TIL-B subtypes in HCC patients

**FIGURE 5:**
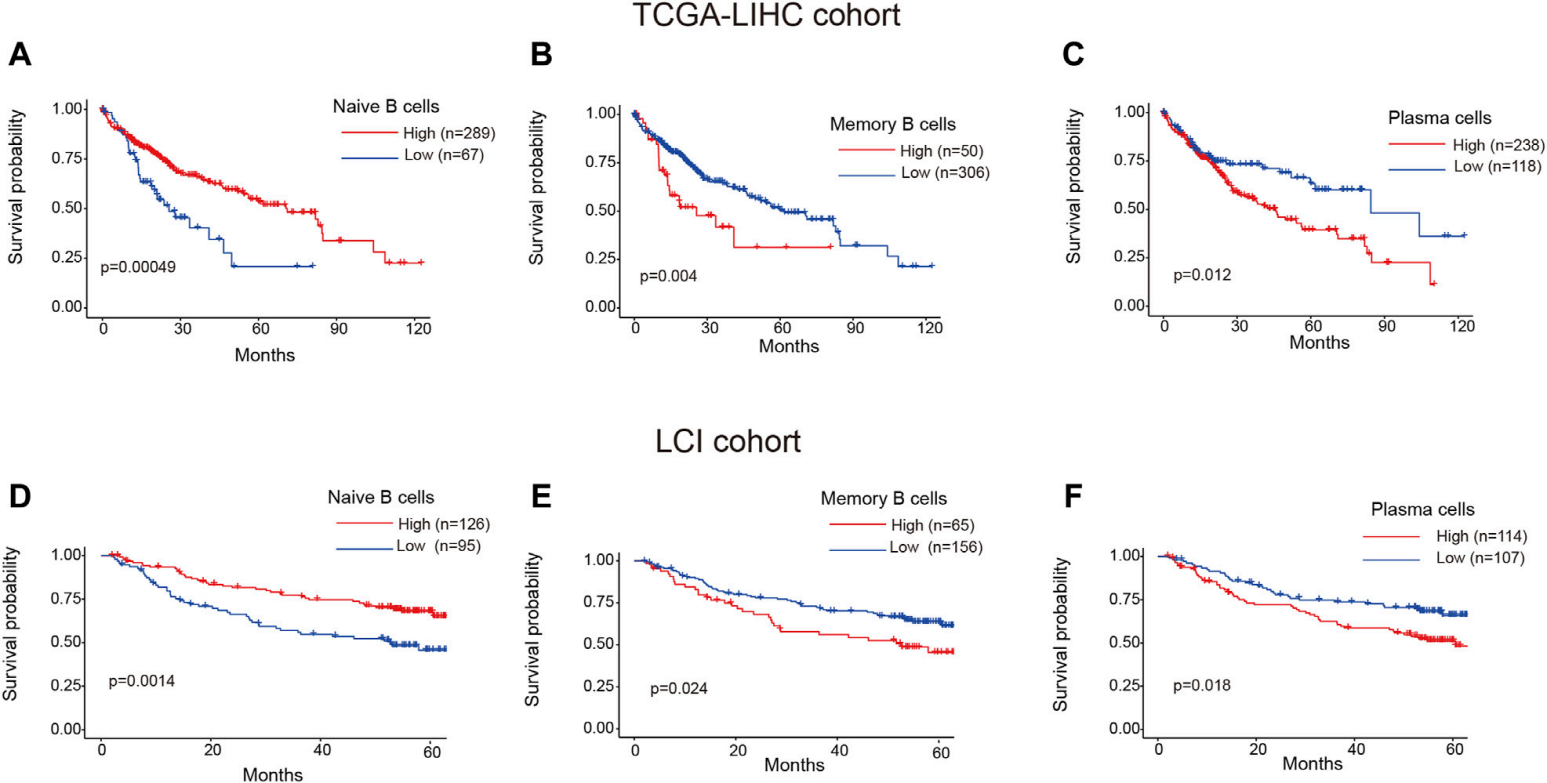
Clinical prognosis of TIL-Bs subtypes in HCC patients. **(A–C)** The prognosis of naïve B cells, memory B cells and plasma cells in TCGA-LIHC cohort. **(D–F)** The prognosis of naïve B cells, memory B cells and plasma cells in LCI cohort.

To investigate the clinical relevance of TIL-B subtypes in HCC patients, we utilized the CIBERSORT algorithm to calculate the ratios of 22 immune cells in both TCGA-LIHC and LCI cohorts. The “surv_cutpoint” function from the Survminer R package was employed to determine the optimal cutoff value for the proportion of naive B cells, memory B cells and plasma cells. Subsequently, patients were stratified into two groups based on this cutoff value. Surprisingly, distinct TIL-B subpopulations exhibited varying clinical outcomes in HCC patients. Specifically, patients with a higher infiltration of naïve B cells demonstrated improved overall survival in both TCGA-LIHC and LCI cohorts (Figures 5A, D). Conversely, patients with elevated levels of memory B cells and plasma cells displayed a reduced overall survival probability (Figures 5B, C, E, F). These findings emphasize the substantial heterogeneity within TIL-B populations and underscore the need to further explore the specific role of TIL-Bs in anti-tumor immunity at a finer resolution.

### Construction and validation of plasma cell marker-based prognostic model

**FIGURE 6:**
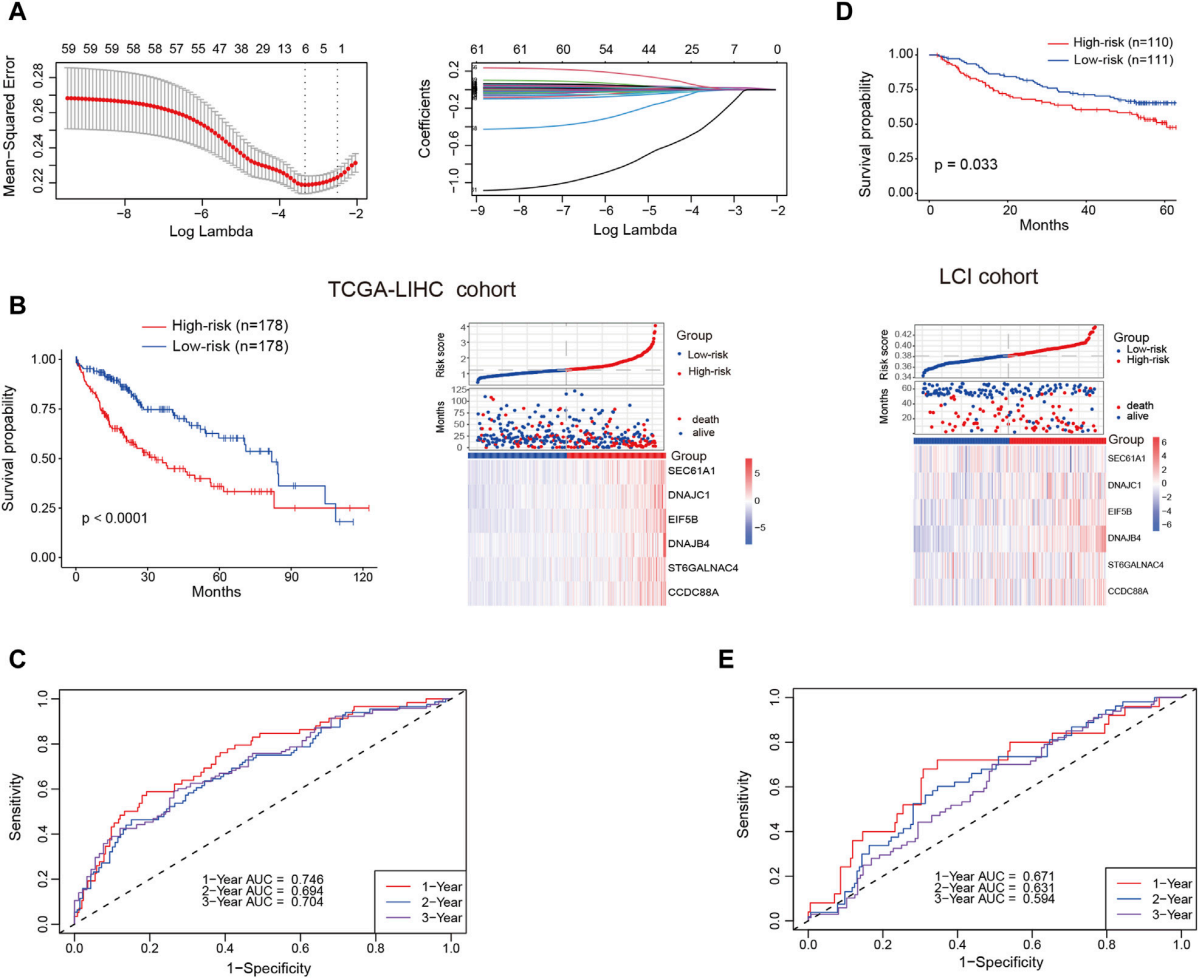
Prognostic risk model construction and validation. **(A)** Identification of the most representative plasma cell marker genes through LASSO analysis. **(B)** Comparison of overall survival (OS) and expression levels of candidate genes between high-risk and low-risk patients in the training cohort (TCGA-LIHC) **(C)** Area under the curve (AUC) values for 1-year, 2-year and 3-year survival in the training cohort. **(D)** Comparison of OS and expression levels of candidate genes between high-risk and low-risk patients in the test cohort (LCI). **(E)** AUC values for 1-year, 2-year and 3-year survival in the test cohort.

Our analysis unveiled a notable enrichment of plasma cells in tumor tissues, which was associated with unfavorable clinical outcomes. Genes highly expressed in plasma cells are, on one hand, associated with plasma cell identification, and on the other hand, they may serve as functionally relevant genes. We deemed it valuable to construct a plasma cell marker-based prognostic risk model that elucidates the impact of plasma cells on HCC patients. Initially, we identified 242 plasma cell marker genes using the FindAllMarkers function within the Seurat. Subsequently, we intersected these marker genes with expression matrix of the TCGA-LIHC cohort. Subsequent univariable Cox regression analysis identified 61 potentially prognostic genes within the TCGA-LIHC cohort, with 59 of them considered as risk factors. Given the adverse role attributed to plasma cells, we exclusively selected risk genes for LASSO analysis. Ultimately, this process led to the identification of six candidate genes (SEC61A1, DNAJC1, EIF5B, DNAJB4, ST6GALNAC4 and CCDC88A) for constructing the prognostic model (Figure 6A). All these six genes were highly expressed by plasma cells (Supplementary Figures S4A, B). Then the identified genes were utilized to construct a prognostic risk model using multivariate Cox regression analyses, employing the following formula: riskscore = SEC61A1_exp_*0.000811515+DNAJC1_exp_*0.009154278+EIF5B_exp_*0.012715447+DNAJB4_exp_*0.018562242+ST6GALNAC4_exp_*0.013184291+CCDC88A_exp_*0.009150427, where gene_exp_ represented the expression level of each gene. Subsequently, the risk score was calculated for patients in the training cohort (TCGA-LIHC) and the test cohort (LCI). Based on the median risk score, HCC patients were categorized into high-risk and low-risk groups, with those in the high-risk group displaying a less favorable prognosis in the training cohort (Figure 6B). The receiver operating characteristic curve (ROC) curve demonstrated that the AUC for 1-year, 2-year and 3-year survival was 0.746, 0.694, and 0.704, respectively (Figure 6C). Likewise, patients classified as high-risk in the test cohort were linked to an adverse prognosis, exhibiting an AUC of 0.671, 0.631, and 0.594 for 1-year, 2-year, and 3-year survival, respectively (Figures 6D, E). These findings indicate the robust predictive capability of the prognostic risk model in determining the clinical outcomes of HCC patients.

### Improved immunotherapy response in low-risk patients

**FIGURE 7:**
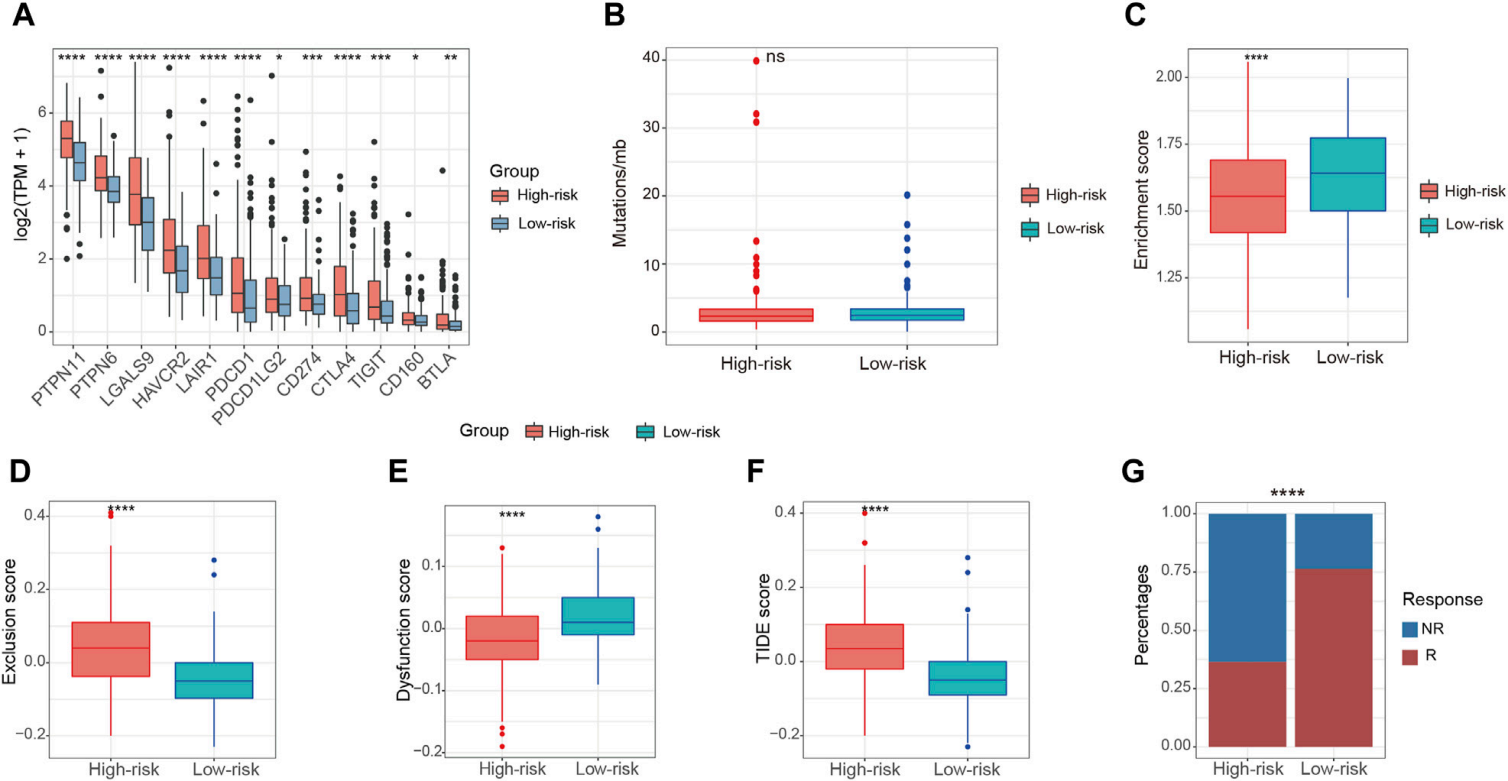
Relationship between risk score and immunotherapy in the TCGA-LIHC cohort. **(A)** The expression of immune checkpoint factors between high-risk and low-risk patients. **(B)** The levels of TMB between high-risk and low-risk patients. **(C)** The enrichment score of TLS signatures between high-risk and low-risk patients. **(D,E)** The exclusion score and dysfunction score between high-risk and low-risk patients according to TIDE algorithm. **(F)** The TIDE score between high-risk and low-risk patients according to TIDE algorithm **(G)** The composition of immunotherapy efficacy between high-risk and low-risk patients predicted by TIDE algorithm. ns, not significant, *p < 0.05, **p < 0.01, ***p < 0.001, ****p < 0.0001.

Immunotherapy stands as a paramount therapeutic approach in the treatment of HCC, especially in scenarios where surgical intervention is not a viable option. To assess the immunotherapeutic effects on patients in these two groups, we examined several predictors of immunotherapy response. These predictors included the expression of immune checkpoint factors, tumor mutation burden (TMB), and the presence of TLS. High-risk patients were found to exhibit increased expression of immune checkpoint factors in comparison to those in the low-risk group (Figure 7A). This observation suggests an immunosuppressed microenvironment, which may contribute to the poorer overall survival outcomes observed in the high-risk patients. Nonetheless, the high-risk and low-risk groups demonstrated comparable levels of TMB (Figure 7B). Recent rigorous investigations have unveiled a constructive association between the increased presence of TLS and a heightened response to ICI (Schumacher and Thommen, 2022). Notably, relative to the high-risk group, the enrichment score of TLS signatures was higher in the low-risk group (Figure 7C). The application of the TIDE algorithm allowed for the evaluation of disparities in immunotherapy response among patients in both groups. The findings indicated a more elevated exclusion score and a correspondingly reduced dysfunction score within the high-risk patients when contrasted with the low-risk patients (Figures 7D, E). Conversely, the low-risk group exhibited a lower TIDE score and a greater possibility of a positive response to immunotherapy (Figures 7F, G).

### Better chemotherapy sensitivity in high-risk patients

**FIGURE 8:**
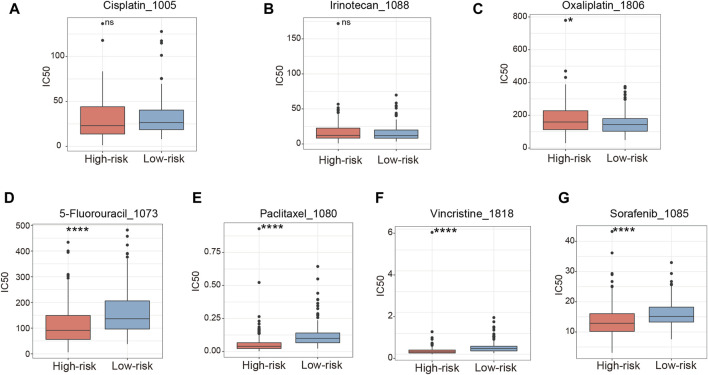
Relationship between risk score and chemotherapy in the TCGA-LIHC cohort. **(A–G)** The sensibility score of cisplatin, irinotecan, oxaliplatin, 5-fluorouracil, paclitaxel, vincristine and sorafenib between high-risk and low-risk groups. ns, not significant, *p < 0.05, ****p < 0.0001.

Chemotherapy, although characterized by limited efficacy in the treatment of HCC, remains a crucial therapeutic avenue for disease management, especially when other treatment modalities prove ineffective. IC50 of common chemotherapeutic drugs and targeted drugs for treating HCC were estimated for each patient in TCGA-LIHC cohort. Our data indicated that there were no notable variations in the responsiveness to cisplatin and irinotecan between the high-risk and low-risk groups (Figures 8A, B). Conversely, the low-risk group demonstrated a greater sensitivity to oxaliplatin, as reflected by a lower IC50, in comparison to the high-risk group (Figure 8C). However, the IC50 of 5-fluorouracil, paclitaxel and vincristine was found to be diminished in the high-risk group when contrasted with the low-risk group (Figures 8D–F). Sorafenib is an established first-line targeted therapy for advanced HCC (Tang et al., 2020). Compared to the low-risk group, patients with a high-risk score were inclined to exhibit sensitivity to sorafenib (Figure 8G). These observations indicate that despite high-risk patients demonstrating lower efficacy in immunotherapy, they may derive benefit from chemotherapeutic drugs and targeted drugs.

### Robust predictive accuracy of plasma cell marker-based prognostic model

**FIGURE 9:**
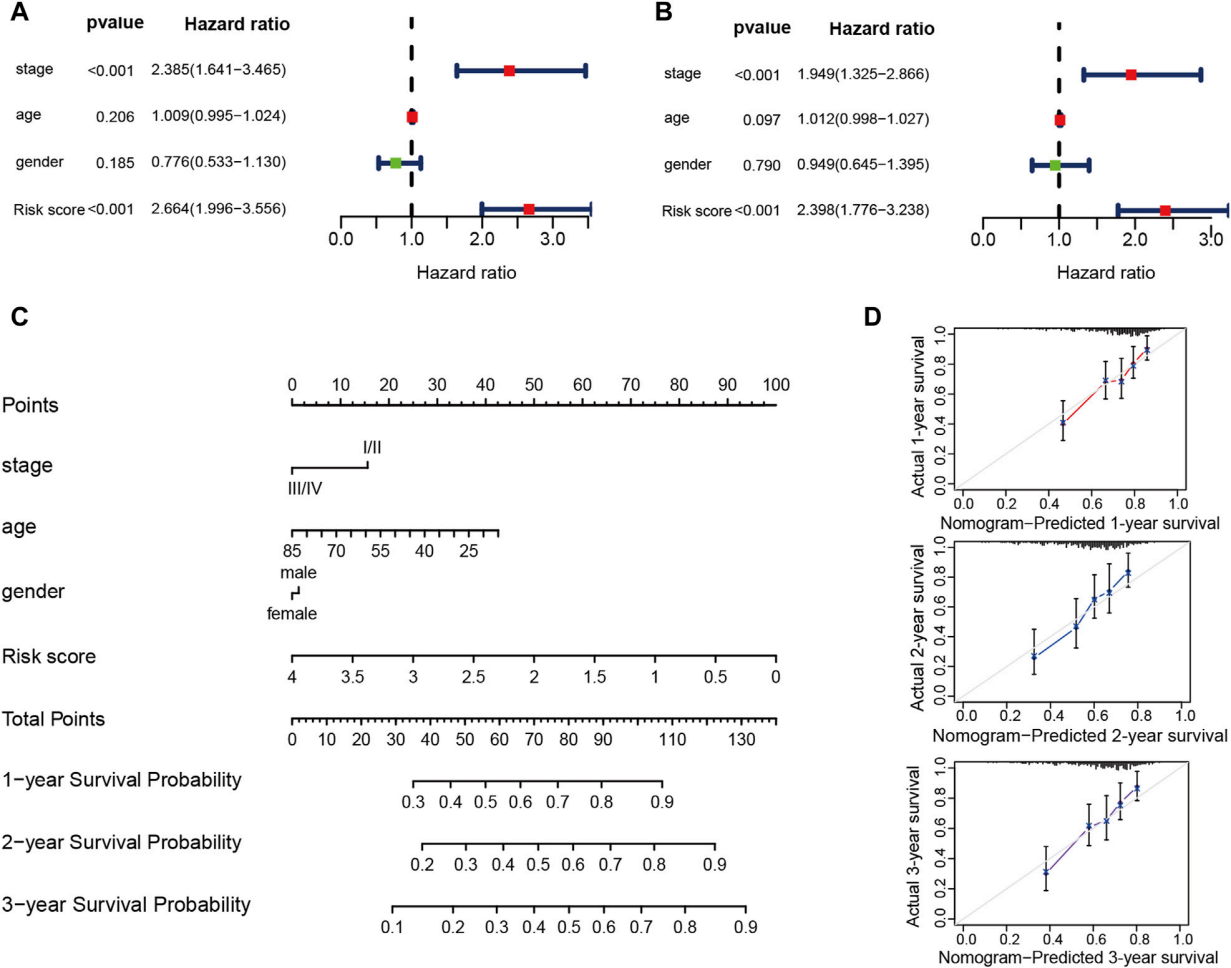
The prognostic risk model shows robust predictive accuracy in TCGA-LIHC cohort. **(A,B)** The results of the univariate and multivariate Cox regression analyses regarding OS in the TCGA-LIHC cohort. **(C)** The nomogram showing the 1-, 2- 3-year OS of age, gender, stage and risk score. **(D)** The Calibration plots showing the predicted and actual OS for 1-, 2- 3-year survival probabilities in TCGA-LIHC cohort.

The risk score’s ability in independently predicting overall survival (OS) was estimated through univariate and multivariate Cox regression analyses. Both univariate (HR = 2.664, 95% CI = 1.996–3.556, *p* < 0.001) and multivariate (HR = 2.398, 95% CI = 1.776–3.238, *p* < 0.001) Cox regression analyses (Figures 9A, B), unequivocally confirmed the risk score’s status as an independent prognostic predictor. Moreover, we performed a nomogram analysis that incorporates risk score, disease stage, patient age, and gender to predict overall survival probabilities. The nomogram efficiently predicted the probabilities of overall survival at 1, 2, and 3 years using the prognostic risk model. The C-index of this nomogram model was calculated to be 0.702 (Figure 9C). The calibration curve, which compared the nomogram-predicted OS probabilities to the actual probabilities, demonstrated a satisfactory overlap, signifying optimal predictive accuracy (Figure 9D).

## Discussion

The immune cells in the TME have been demonstrated to exert significant impacts on carcinogenesis and profoundly influence the clinical outcomes of cancer patients (Zhang and Zhang, 2020). Within the realm of cancer immunotherapy, the involvement of T cells and myeloid cells has been extensively explored using scRNA-seq, the importance of B cells in cancer immunity has also been acknowledged (Zheng et al., 2017; Zhang Q. et al., 2019; Patil et al., 2022). To date, scRNA-seq has been utilized for the examination of the phenotypic and functional diversity of TIL-Bs across diverse cancer types, encompassing melanoma, breast cancer, colorectal cancer, non-small cell lung cancer, endometrial cancer and early-stage lung adenocarcinoma (Griss et al., 2019; Chen et al., 2020; Hu et al., 2021; Hao et al., 2022; Horeweg et al., 2022; Patil et al., 2022; Xia et al., 2023). However, the single-cell landscape of TIL-Bs in HCC remains unexplored. In this research, we performed an extensive examination of the subtypes and functional characteristics of TIL-Bs in HCC. We elucidated the intercellular communication pathways that exist between TIL-Bs and other cells in the TME. Furthermore, our findings shed light on the relationship between therapy efficacy and a prognosis risk model constructed with plasma cell marker genes, which could potentially offer personalized treatment options for HCC patients with varying degrees of plasma cell infiltration.

Our data revealed the existence of five distinct subpopulations of TIL-Bs in HCC, each with unique infiltration characteristics. Notably, in tumor tissues, B cells displayed a tendency to differentiate into plasma cells, resulting in the enrichment of plasma cells in tumor tissues. While B cells differentiated, genes linked to T cell differentiation and activation were downregulated, a phenomenon that may contribute to the plasma cells’ adverse prognosis. Furthermore, naïve B cells and memory B cells exhibited distinct functions in terms of clinical outcomes, emphasizing the need for a higher-resolution analysis of TIL-Bs.

The interactions between immune cells within the TME are critical determinants of the direction of tumor immunity, influencing whether it favors tumor promotion or tumor suppression (Zhang and Zhang, 2020). We observed an interaction between Plasma cells-JCHAIN and Macrophages-SPP1 through the GAS6-MERTK pathway, which previously implicated in the development of various cancer types (Ammoun et al., 2014; Chiu et al., 2015). Interestingly, B cells could activate both CD8-XCL1 and CD4-FOXP3 through the TNFSF9-TNFRSF9 pathway, indicating that B cells performance is shaped by the binding strength and differential presence of T cell subtypes. Earlier research has demonstrated the participation of macrophages in B cell differentiation leading to plasma cell formation (Wei et al., 2019). In our study, we indeed observed interactions between macrophages and B cells through TNFSF13B-TNFRSF13B/TNFRSF13C and TNFSF13-TNFRSF13B signaling pathways, which are instrumental in guiding B cell differentiation into plasma cells (Vincent et al., 2013). It has previously been shown that the location of TIL-Bs within the tumor microenvironment can have significant implications for cancer outcomes. TIL-Bs located in the cancer nest have been associated with cancer elimination, while those located at the cancer edge are associated with early cancer recurrence (Liu et al., 2015; Zhang Z. et al., 2019; Wei et al., 2021). In colorectal cancer (CRC), plasma cells can be recruited to the cancer nest by cancer cells through CCL28-CCR10 signaling, thereby exerting anti-tumor effects (Xia et al., 2023). In our study, we observed that cancer cells were unable to recruit plasma cells to the cancer nest, which could contribute to the unfavorable prognosis associated with increased plasma cell infiltration in HCC. In the context of TLS, the recruitment of B cells is primarily mediated by the chemokine CXCL13, secreted by T follicular helper cells (Tokunaga et al., 2019). Consistent with previous studies in lung cancer and melanoma, our study demonstrated that the CD8-XCL1 cluster secreted CXCL13 and recruited B cells via the CXCL13-CXCR5 signaling pathway to form TLS, thereby promoting anti-tumor immunity (Thommen et al., 2018; Li et al., 2019; Workel et al., 2019).

Considering the upregulation of plasma cells and their association with unfavorable prognosis in tumor tissues, we selected genes highly expressed by plasma cells to establish a prognostic risk model. Consistent with the poor prognosis associated with plasma cells, 59 out of 61 genes associated with prognosis were identified as risk factors. After conducting LASSO analysis, we identified SEC61A1, DNAJC1, EIF5B, DNAJB4, ST6GALNAC4 and CCDC88A as the most representative gene markers for constructing a prognostic risk model. Our findings substantiated the risk score’s standing as an independent prognostic indicator for overall survival. Additionally, a relationship was identified between the risk score and the effectiveness of HCC treatment. Elevated expression of immune checkpoint genes was observed in the high-risk group, but they were predicted to derive less benefit from immunotherapy, possibly due to the immune-excluded phenotype. Interestingly, those classified in the high-risk group were more inclined to experience benefits from 5-fluorouracil, paclitaxel, and vincristine. All these chemotherapeutic drugs are associated with the cell cycle, with paclitaxel and vincristine acting as microtubule inhibitors, while 5-fluorouracil primarily inhibits DNA synthesis during the S phase (Yano et al., 2020). High-risk patients exhibited higher expression of cell cycle-associated genes (Supplementary Figure S5), potentially explaining their sensitivity to chemotherapy.

## Conclusion

In conclusion, this study unveiled the characteristics of TIL-Bs and the distinct prognostic implications of TIL-Bs subtypes in HCC patients. We underscored the significance of different interactions between TIL-Bs and other cells in shaping the HCC TME and anti-tumor immune response. Furthermore, the prognostic risk model based on plasma cell markers holds promise for selecting appropriate treatment strategies for HCC patients.

## Data Availability

The datasets presented in this study can be found in the Genome Sequence Archive at the National Genomics Data Center in Beijing, China (https://ngdc.cncb.ac.cn/gsa-human/) under the serial number HRA004750. The code used for all processing and analysis can be obtained from the following link: https://github.com/liyuanqixyz/CODE/blob/main/CODE.R.

## Supplementary material

SUPPLEMENTARY FIGURE S1 The landscape of infiltrating T cells in HCC. **(A)** UMAP showing subtypes of T cells. **(B)** Alluvial diagram showing percentages of T cell subtypes between tumor and normal liver tissues. **(C)** Violin plot showing expression of conventional marker genes in subtypes of T cells. **(D)** Heatmap showing expression of functional genes in subtypes of T cells. **(E,F)** Dot plot showing expression of co-inhibitory molecules **(E)** and co-stimulatory molecules **(F)** in subtypes of T cell.

SUPPLEMENTARY FIGURE S2 The landscape of infiltrating myeloid cells in HCC. **(A)** UMAP showing subtypes of myeloid cells. **(B)** Alluvial diagram showing percentages of myeloid cell subtypes between tumor and normal liver tissues. **(C)** Violin plot showing expression of conventional marker genes in subtypes of myeloid cells. **(D)** Dot plot showing expression of myeloid-derived suppressor cells (MDSC) marker genes in subtypes of myeloid cells. **(E)** Dot plot showing expression of TAMs marker genes in subtypes of myeloid cells.

SUPPLEMENTARY FIGURE S3
**(A)** Dot plot showing the increased signaling ligand-receptor pairs between TIL-Bs and T cells in tumor tissues. **(B)** Dot plot illustrating the increased signaling ligand-receptor pairs between TIL-Bs and myeloid cells in tumor tissues.

SUPPLEMENTARY FIGURE S4
**(A)** Violin plot showing the expression of six candidate genes among cell types. **(B)** Dot plot illustrating the expression of six candidate genes among cell types.

SUPPLEMENTARY FIGURE S5 Enrichment analysis of genes highly expressed by high-risk patients. **(A,B)** the result of GO and KEGG enrichment analysis of genes highly expressed by high-risk patients.
