# Supplementary figures and images for: Identification of prognostic risk model based on plasma cell markers in hepatocellular carcinoma through single-cell sequencing analysis
Source: Front Genet. 2024 May 27;15:1363197. doi: 10.3389/fgene.2024.1363197 (PMC11163121; doi:10.3389/fgene.2024.1363197)

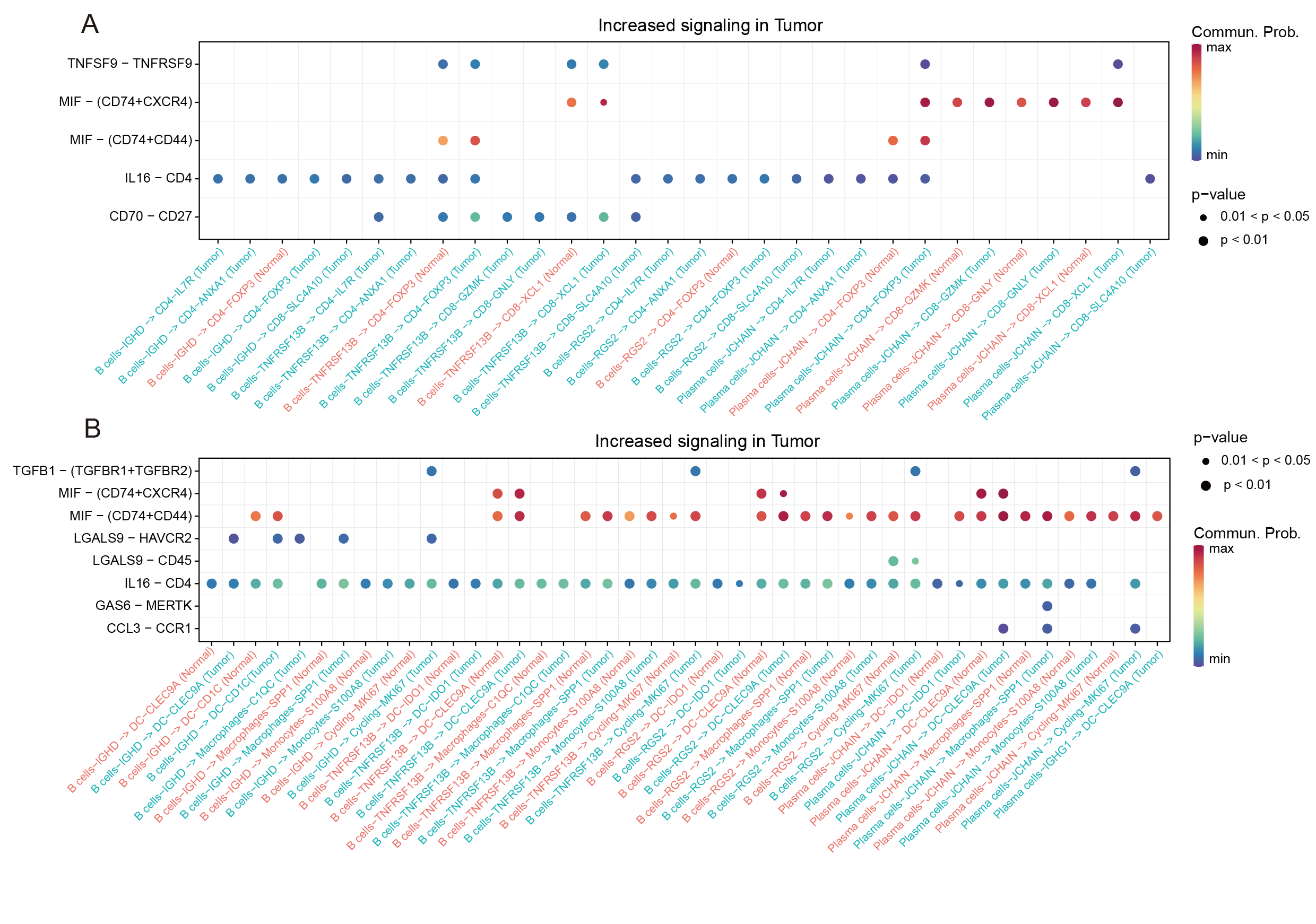

Supplement: Supplementary file 3 [file Image3.TIF]

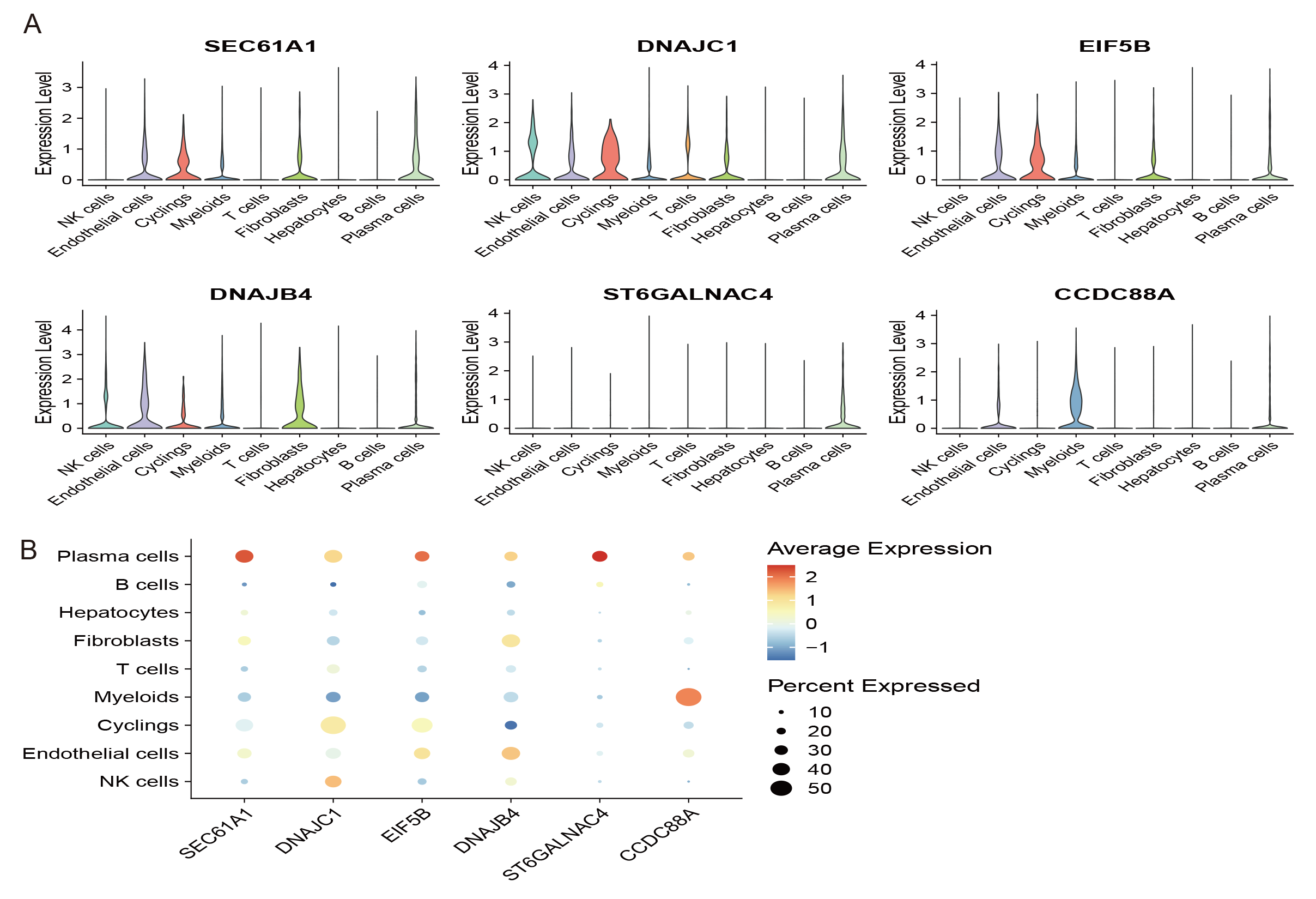

Supplement: Supplementary file 4 [file Image4.TIF]

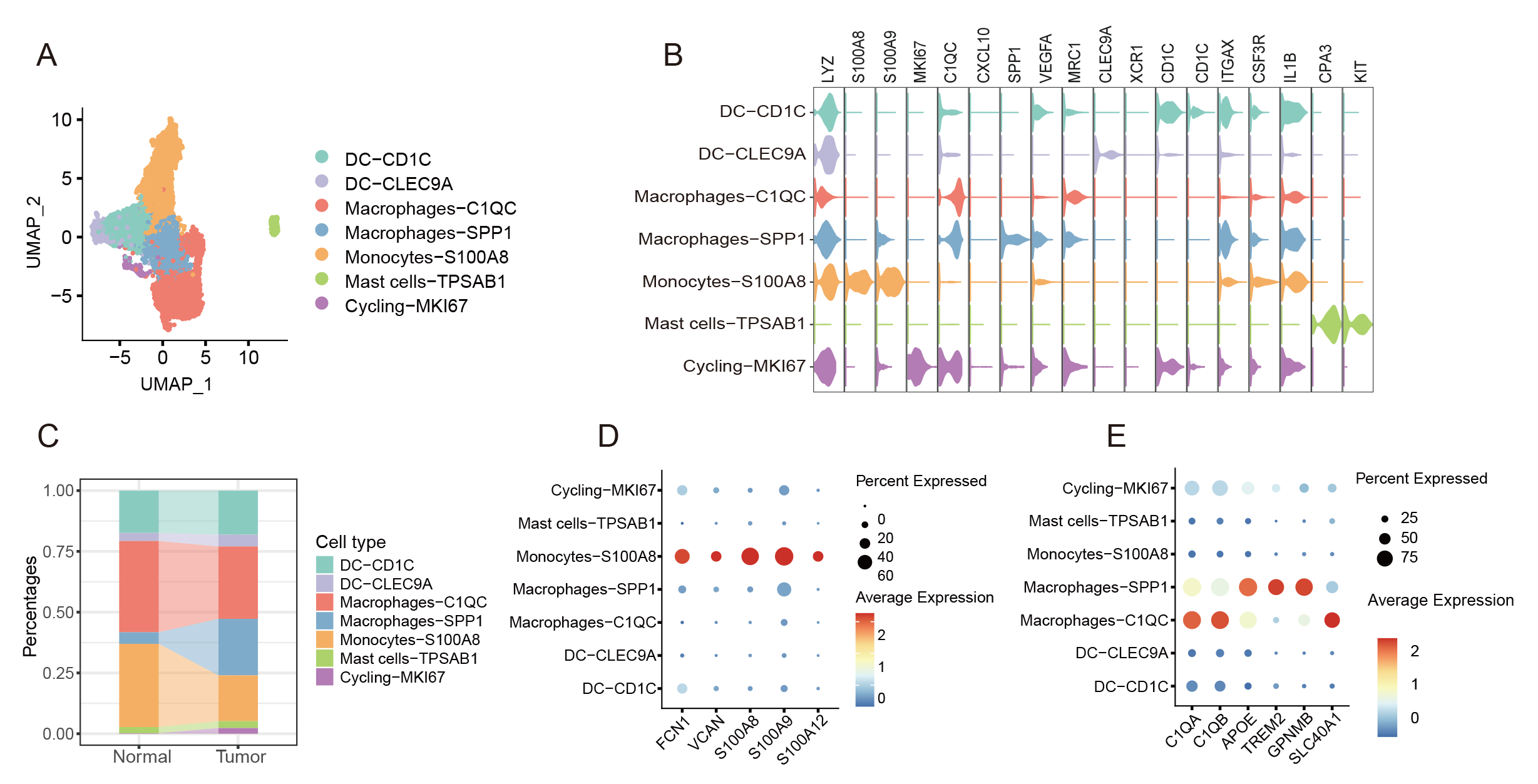

Supplement: Supplementary file 5 [file Image2.TIF]

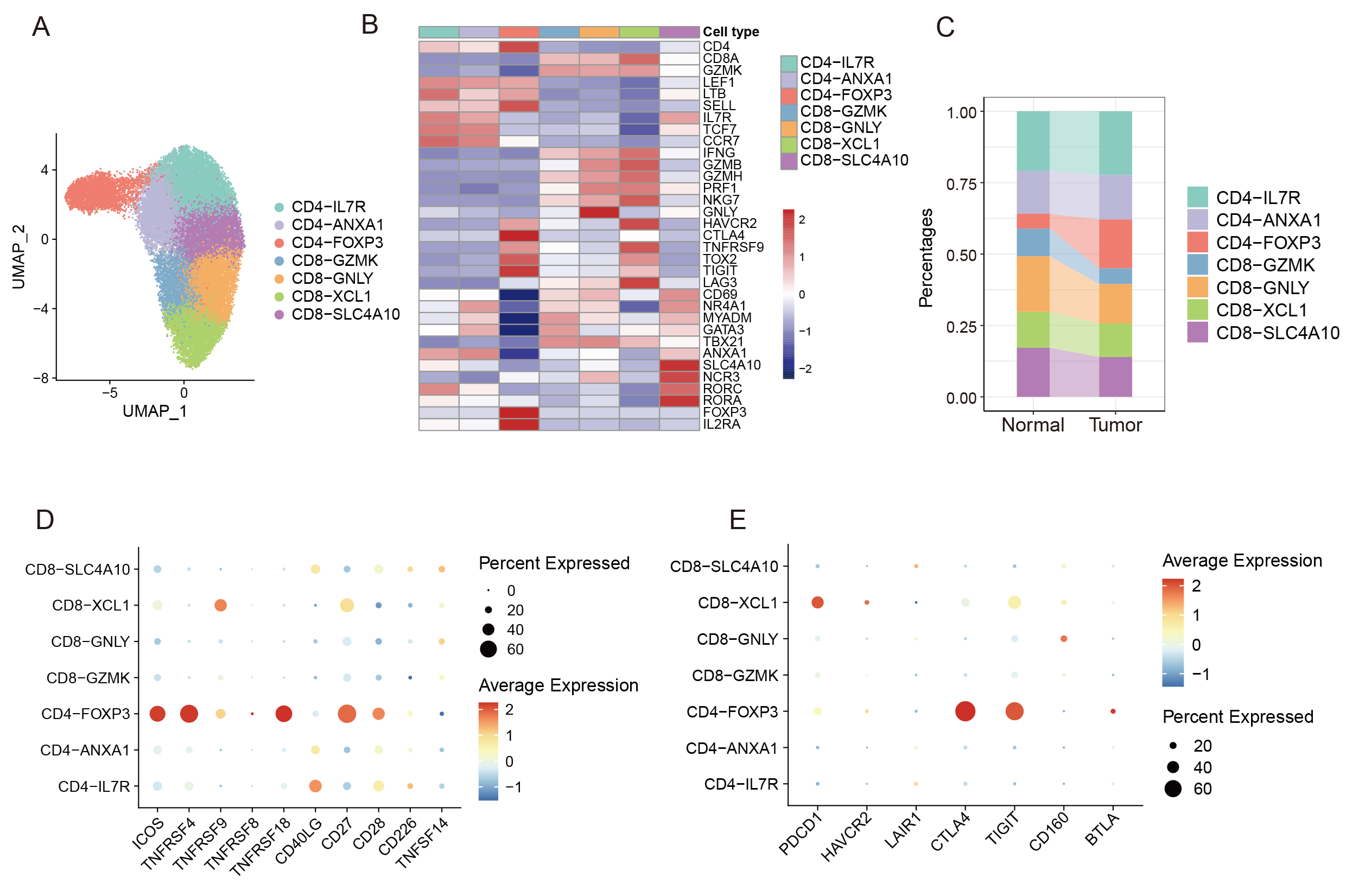

Supplement: Supplementary file 6 [file Image1.TIF]

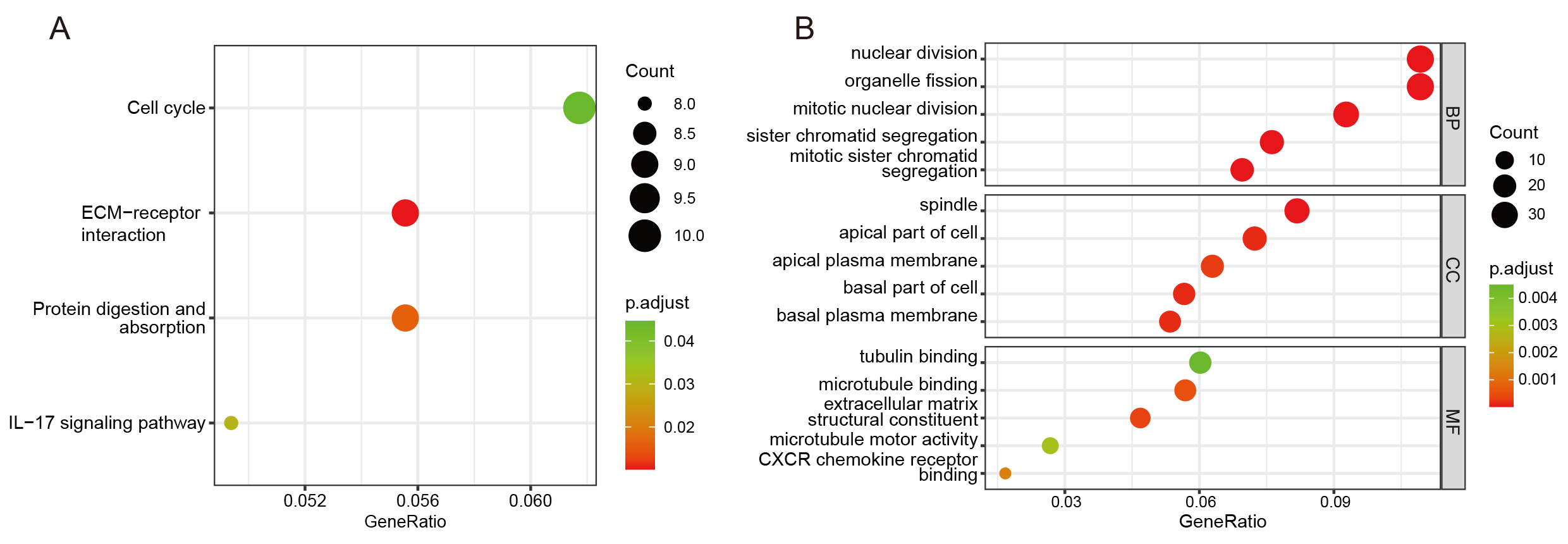

Supplement: Supplementary file 8 [file Image5.TIF]
